# A Bridge Between East and West: Dr. Johan Louis Schlimmer’s Medical Contributions in Qajar Iran (1819-1881)

**DOI:** 10.7759/cureus.69015

**Published:** 2024-09-09

**Authors:** Kamran Shirbache, Amirreza Liaghat, Hamidreza Namazi

**Affiliations:** 1 Orthopedics, Robert Debré Hospital, Paris, FRA; 2 Immunology, Université Bordeaux Montaigne, Bordeaux, FRA; 3 Medical Ethics, School of Medicine, Medical Ethics and History of Medicine Research Center, Tehran University of Medical Sciences, Tehran, IRN

**Keywords:** dar al-funun, historical vignette, iran modern medicine, johan louis schlimmer, qajar public health

## Abstract

Dr. Johan Louis Schlimmer (1819-1881), a Dutch physician, played a transformative role in the development of modern medicine in Qajar-era Iran. Arriving in Iran driven by a deep passion for Eastern culture, Schlimmer’s contributions extended far beyond the confines of traditional medical practice. His work in medical education, particularly at the prestigious Dar al-Fonun, laid the foundation for the modernization of Iranian healthcare. Schlimmer’s efforts to bridge the gap between Western and Iranian medical knowledge are epitomized by his collaboration on the Persian medical dictionary, commonly known as *Schlimmer's Dictionary*, and his seminal work, *Terminologie Médico-Pharmaceutique et Anthropologique Française-Persane*. These texts not only expanded the Persian medical lexicon but also integrated contemporary Western medical concepts with traditional Iranian practices. Schlimmer’s approach, which emphasized the indigenization of medical knowledge, made complex medical phenomena accessible to Iranian practitioners and laid the groundwork for the advancement of public health in Iran. His legacy, marked by a commitment to cultural and scientific exchange, continues to resonate in the field of Iranian medical history. This article explores the multifaceted contributions of Dr. Schlimmer, highlighting his enduring impact on the evolution of healthcare in 19th-century Iran.

## Introduction and background

Dr. Johann Louis Schlimmer, known in Iran as Hakim Schlimmer Flemingy, was a Dutch physician who made significant contributions to the advancement of modern medicine in Iran during the Qajar era (13th century AH, 1796-1925) [1]. A key figure in the medical history of Iran, Schlimmer's influence has endured long after his time, with his work continuing to serve as a reference in Iranian medical education. Born in 1819 (1234 AH) in Rotterdam, Netherlands, Schlimmer received his medical education at Leiden University [2]. His profound interest in Eastern culture led him on a journey to the East, where he gained invaluable experience through research and medical practice in Syria and Iraq [3].

Around 1844, Schlimmer arrived in the province of Gilan in northern Iran [1]. However, it was the esteemed reputation of Dar al-Fonun, a pioneering institution established by Amir Kabir, the prime minister of Iran, to promote the education of modern sciences, that ultimately drew Schlimmer to Tehran. Dar al-Fonun, founded in 1851, was created in response to the urgent need for military modernization, which increasingly relied on the integration of contemporary scientific knowledge [4]. Schlimmer became one of the first instructors at this institution, following in the footsteps of Dr. Jakob Eduard Polak (1818-1891), an Austrian physician who played a crucial role in introducing modern medicine to Iran [3,5].

Schlimmer's contributions to Iran were far-reaching, and his role extended well beyond that of a European medical lecturer. Through years of dedicated research and medical practice, he earned considerable respect and recognition. The following sections will delve into the invaluable contributions of this pioneering physician and the lasting legacy he left on Iranian medicine.

## Review

A bridge between two cultures

Europe and the Middle East have long stood as epicenters of intellectual and cultural development, each offering unique contributions to the history of humanity. For centuries, cultural envoys from both regions have endeavored to bridge these two worlds, fostering mutual understanding and connection [6]. This endeavor, however, has always required a nuanced approach, given the distinct ideological and social differences that define each culture. The field of medical education, along with the transfer of medical knowledge and technology, was no exception to this intricate and delicate process [7].

Dr. Johann Louis Schlimmer was deeply interested in the historical medical texts of Persia, such as the “Khwarazmshahi Reserve.” Recognizing the importance of indigenizing medical science, he understood that his work needed to be culturally and historically relevant to the society he served. To achieve this goal, Schlimmer immersed himself in the rich cultural fabric of Iran, enhancing his knowledge and experience by traveling to various regions of the country [2]. His explorations were not merely academic; they involved studying specific endemic infectious diseases, learning from local communities and their traditional medical practices, and ultimately educating the population to improve their health conditions [8]. This holistic approach distinguished Schlimmer from his contemporaries.

**Figure 1:**
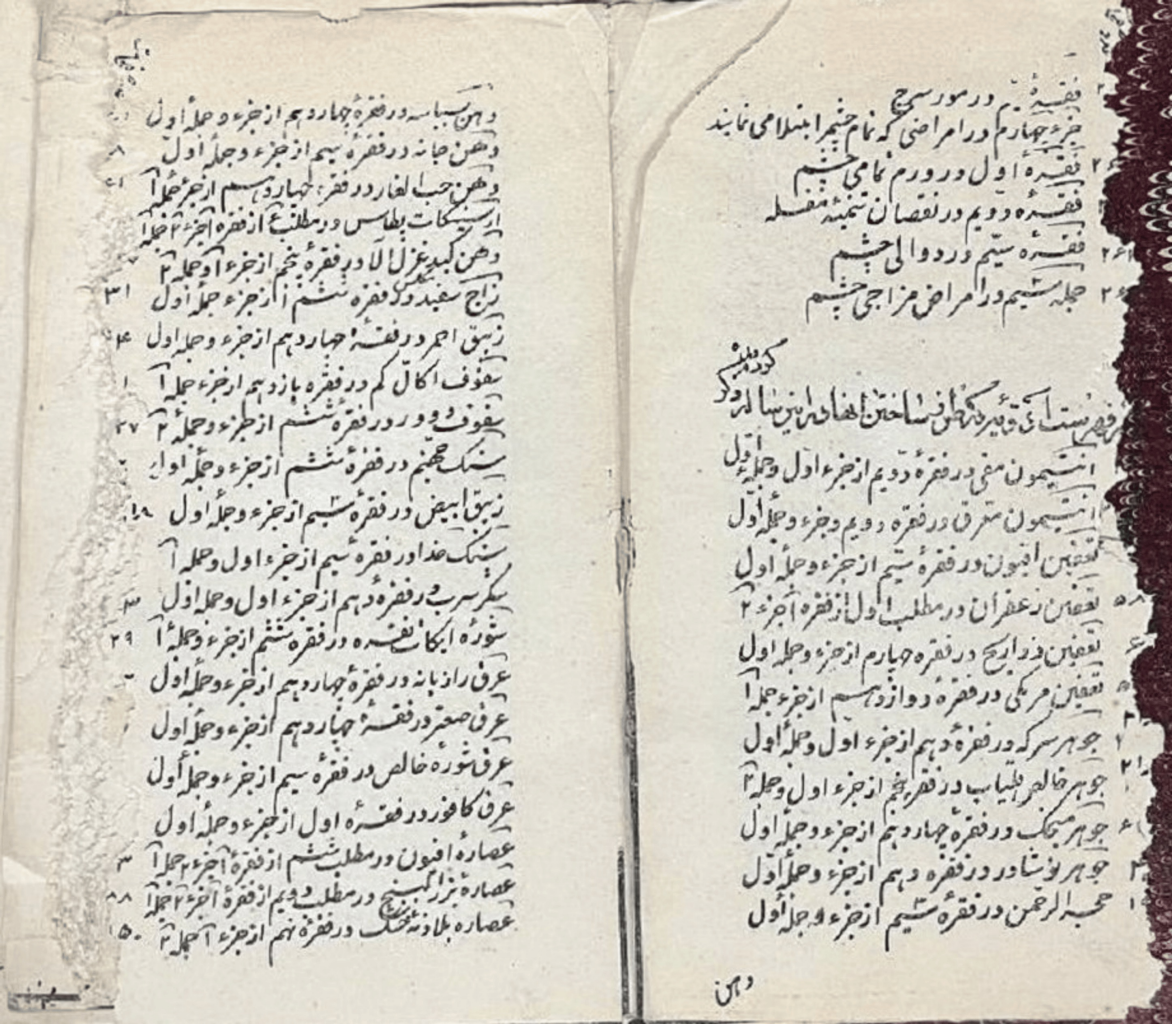
An original text book of Louis Schlimmer This image is courtesy of Hamidreza Namazi, the current head of the National Museum of the History of Medical Sciences in Iran and the corresponding author of this article

For example, during his time in Gilan, Schlimmer encountered families afflicted by leprosy, a condition that led to their ostracization by society. Having never treated leprosy patients before, Schlimmer brought these individuals to the city, where he meticulously studied and treated them. This hands-on approach to medical research was emblematic of Schlimmer’s commitment to understanding and addressing health issues in a practical, patient-centered manner. His work not only improved the lives of leprosy patients but also aimed to alter the public’s negative perceptions and feelings of disgust toward them. His educational books on leprosy were a direct outcome of these efforts, and his successful practices in treating leprosy, along with his anatomical research on the dissection of the dicephalic calf in Rasht, earned him considerable respect and recognition in Gilan (Figure 1) [1,3].

Schlimmer’s commitment to medical science also took him to Baluchistan and Bandar Abbas, where he studied diseases such as guinea worm disease (piyuk, rešta) and smallpox. In Baluchistan, he observed the traditional practice of treating smallpox with secretions from cows infected with the disease. While introducing the local population to scientifically sound methods, Schlimmer also showed respect for their traditional practices, recognizing the value of their methods as an early form of vaccination [1].

As a professor at Dar al-Fonun, where modern medicine in Iran was closely tied to the military, Schlimmer was granted the rank of colonel [9]. This military affiliation provided him with opportunities to embark on missions to remote regions such as Kurdestan, Kashan, and Kerman. These journeys not only expanded his knowledge of endemic diseases and regional health challenges but also underscored the importance of public health. His direct engagement with diverse communities and firsthand experiences with their living conditions led him to recognize the critical role of public health in promoting societal well-being [3].

Like many European physicians in Iran, who primarily served the royal court, Schlimmer served as the royal family's physician [10]. However, he also maintained a private clinic where he treated ordinary citizens, including the poor. His dual exposure to both the affluent and impoverished segments of society deepened his understanding of the fundamental issues affecting public health [2]. In an era when Iran, like many other regions, was plagued by rampant infectious diseases such as leprosy, plague, syphilis, smallpox, recurrent fever, cholera, elephantiasis (dāʾ al-fil), and tinea (kačali), the field of epidemiology was scarcely understood, and the significance of public health in controlling the spread of infections was largely unrecognized. Most physicians focused solely on treating individual patients, with little attention given to broader, systemic efforts to curb the incidence of these diseases [5,11].

However, Schlimmer’s extensive experiences in various local practices and his inquisitive spirit led him to consider public health as a critical factor in improving the health conditions of society. His contributions to public health in Iran were pioneering, laying the foundation for future efforts to enhance population health through a comprehensive understanding of public health principles. His legacy continues to influence the ongoing work to improve the well-being of communities through a balanced integration of traditional knowledge and modern scientific practices [12].

Establishing a unified medical language for optimal education

In any meaningful cultural exchange, establishing a common language is essential for fostering constructive relationships. Dr. Johann Louis Schlimmer, with his profound understanding of Iran’s cultural and scientific landscape, recognized the importance of this endeavor. To bridge the gap between traditional Iranian medical knowledge and modern Western practices, Schlimmer emphasized the need to focus on the Persian language as a medium for scientific discourse [12].

Schlimmer’s contributions to the Persian medical lexicon began with his collaboration with Mirza Ali Akbar Khan Nafisi, leading to the compilation of the Persian medical dictionary, widely known as "Schlimmer's Dictionary" [3]. This dictionary played a crucial role in expanding the capacity of the Persian language to articulate complex medical concepts, thus enhancing its relevance in the modern scientific context. Schlimmer’s approach was marked by a commitment to indigenization, ensuring that medical knowledge became more accessible to Iranian practitioners. He adeptly integrated everyday language with formal scientific terminology, using familiar terms like "hand" and "shoulder" alongside the more technical "distal upper limb" and "proximal upper limb." This innovative simplification made a significant impact on the development of contemporary Persian medical vocabulary [1].

**Figure 2:**
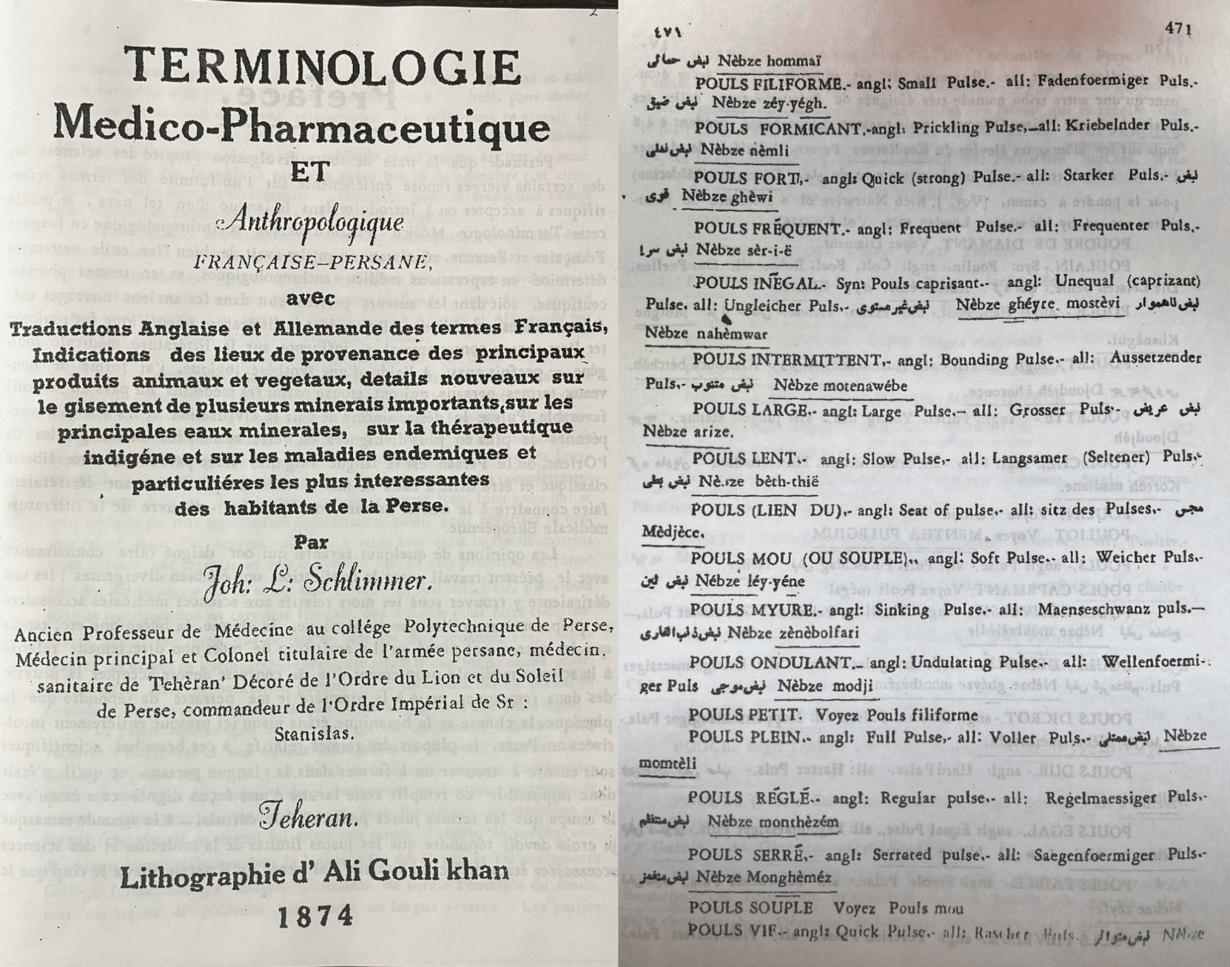
Terminologie Médico-Pharmaceutique et Anthropologique Française-Persane book This image is courtesy of Hamidreza Namazi, the current head of the National Museum of the History of Medical Sciences in Iran and the corresponding author of this article

This groundwork laid the foundation for his esteemed work, “Terminologie Médico-Pharmaceutique et Anthropologique Française-Persane,” published in 1874 (Figure 2). This seminal text represents one of the earliest attempts to harmonize traditional Iranian medical terminology, deeply rooted in the Avicennian tradition, with modern Western medical concepts. The inclusion of "Anthropologique" in the title reflects Schlimmer’s emphasis on the anthropological dimensions of education and treatment throughout his career. The content of the book reveals a remarkable feature: in addition to translations from French to Persian, equivalent terms in German and English are provided, setting it apart from other texts of its time [8,13,14].

Schlimmer’s influence extended far beyond his linguistic contributions. As a prolific author with the highest number of publications in his era, he produced numerous medical texts across various disciplines, many of which became foundational in Iranian medical education (Table1) [3,6,12].

**Table 1:** List of publications of Louis Schlimmer

N.	Title	Field	Date of publication
1	Meftah Al-Khawas [6]	The properties of medicinal herbs	1860
2	Jala-ol-Ayoun [6]	Ophtalmology	1860
3	Serr al-Hekma [6]	Animal anatomy and chemistry	1862
4	Zinat al-Abadan [6]	Dermatology	1862
5	Shafaeyyeh [6]	medical techniques and European classical treatments	1867
6	Terminologie Médico-Pharmaceutique et Anthropologique Française-Persane [6,14]	Encyclopedia of medicine	1874
7	Qavaaed al-Amraaz [6]	General principles of European medicine	1875
8	Asbaab al-Tadwiyeh [6]	Instruction for simple and compound medications	1875
9	Dissection of Neural matter [6]	Neuro-anatomy	1877
10	Montakhab al-Shafaeyyeh [6]	Abstract of European classical treatments	1888
11	Eghrabadin [6]	Pharmacology instruction	1895
12	Amraz al-Sebyan [6]	Pediatric diseases	?
13	Pathology [6]	Pathology	?
14	Tohfat al-Naseri [6]	Pharmacology instruction	?

Education was a cornerstone of Schlimmer’s work. His books were integrated into the curriculum at Dar al-Fonun during his tenure, and they are now regarded as invaluable treasures of Iranian medical history [1]. Dr. Schlimmer began his tenure at Dar al-Fonun as a pharmacology instructor, serving for 10 years. During this period, the influence and tenure of academic figures were often tied to the political leverage of their respective governments within the Qajar court. This political atmosphere may partially explain the brevity of his teaching stint at Dar al-Fonun, although the precise reasons remain unclear and warrant further investigation [9].

Schlimmer also translated his findings and experiences in infectious diseases from French into Persian, delivering lectures and writing manuscripts for students at Dar al-Fonun [15]. His concern for broadening medical education is evident in his abstract books on treatments and his advice to share his lecture notes on cholera and typhus with nonprofessionals during disease crises [6]. Through his teachings, Schlimmer instilled an epidemiological perspective in addressing the challenges of infectious diseases, laying the groundwork for future public health policies aimed at improving urban health and the living conditions of ordinary people [1].

The end of a lifetime journey

While the rich history and culture of Iran have long captivated European scholars, this interest has primarily been the domain of archaeologists, writers, and historians [5]. Rarely have medical or engineering experts ventured to immerse themselves in the intricacies of Iranian society. Dr. Johann Louis Schlimmer, however, stands as a notable exception. Unlike many of his contemporaries at Dar al-Fonun, who arrived in Iran through official channels or external pressures, Schlimmer chose to come to Iran of his own accord, motivated by a profound admiration for Eastern culture [1,8,16]. This decision underscores not only his intellectual curiosity but also his deep affection for the region [12].

Schlimmer's engagement with Iran went far beyond the mere exploration of its cultural and historical treasures. He fully embraced the country as his own, dedicating his life to advancing its healthcare system and improving the well-being of its people [6]. His contributions were multidisciplinary, reflecting his broad interests and skills. In addition to his medical endeavors, Schlimmer served as the Dutch ambassador to Iran for five years, fostering commercial relations between Iran and the Netherlands [17]. He also took the initiative to introduce the manufacturing of glassware at Dar al-Fonun, likely for the production of medical instruments, further illustrating his diverse talents and commitment to the country [3].

His marriage to an Iranian-Armenian woman and his efforts to immerse his children in Iranian culture, including their education at Dar al-Fonun, further attest to his deep connection to the land [6]. Schlimmer’s legacy is enshrined in his contributions, including the compilation of a Persian medical dictionary, the authorship of numerous medical texts in Persian, and his pivotal role in training students at Dar al-Fonun [18]. These efforts stand as enduring testaments to his unwavering dedication to public health and education, significantly improving the quality of medical care in Iran [2].

As one of a small group of European doctors who lived in Iran for over 30 years, Schlimmer passed away in 1881 (1298 AH) and was laid to rest in Akbarabad Presbyterian Cemetery [1,19]. His burial in Iran symbolizes the culmination of a life devoted to a country he came to regard as his own [8].

## Conclusions

Dr. Johan Louis Schlimmer’s life and work exemplify the profound impact a single individual can have on the cultural and scientific evolution of a nation. Through his deep engagement with Iran’s rich cultural heritage and his unwavering commitment to advancing medical knowledge, Schlimmer played a crucial role in the modernization of healthcare during the Qajar era. His linguistic contributions, notably his Persian medical dictionary, equipped Iranian practitioners with essential tools to navigate the complexities of modern medical science.

In today’s world, marked by the challenges of epidemic diseases, conflicts, and preventable deaths among vulnerable populations, the legacy of figures like Johann Louis Schlimmer is more pertinent than ever. His life serves as a powerful reminder of the importance of transcending professional duties to make a lasting impact on the health and well-being of developing societies, particularly in regions like Africa and the Middle East. Schlimmer’s legacy underscores the significance of cross-cultural collaboration, the necessity for localized approaches to healthcare, and the enduring value of a compassionate, holistic approach to medicine. May his life and work inspire contemporary scholars to follow in his footsteps, dedicating themselves to the betterment of humanity.
